# Manganese-Doped Carbon Dots for Sensitive Fluorescence Detection of Ciprofloxacin in Environmental and Pharmaceutical Samples

**DOI:** 10.3390/bios16070357

**Published:** 2026-06-26

**Authors:** Jian Xue, Wenli Fu, Luhang Liu, Qizhong Qin, Jieying Gao, Yingli Li, Anyi Chen

**Affiliations:** 1 College of Public Health, Chongqing Medical University, Chongqing 400016, China; 2019010216@stu.cqmu.edu.cn (J.X.);; 2 Health Management Department, Zunyi Medical and Pharmaceutical College, Zunyi 563006, China

**Keywords:** manganese-doped carbon dots, ciprofloxacin, fluorescence sensing, metal coordination, pond water, pharmaceutical formulations

## Abstract

A simple and sensitive fluorescence sensing method was developed for ciprofloxacin (CIP) determination based on manganese-doped carbon dots (Mn-CDs). The Mn-CDs were synthesized through a one-step hydrothermal method using anhydrous citric acid and manganese chloride tetrahydrate as precursors. The prepared Mn-CDs exhibited good dispersibility, uniform nanoscale morphology, abundant surface functional groups and favorable fluorescence properties. The incorporation of Mn was designed to introduce coordination-related binding sites for CIP, thereby enhancing the interaction between Mn-CDs and CIP. Under excitation at 330 nm, the Mn-CDs showed a pronounced fluorescence enhancement response toward CIP, enabling their use as fluorescent probes for quantitative detection. Under the optimized conditions, the fluorescence intensity increased linearly with CIP concentration over the range of 20 nM–10 μM, with a detection limit of 1.12 nM. The proposed sensing system exhibited satisfactory selectivity toward CIP over various potentially interfering substances and good storage stability. The practicality of the method was further verified by analysis of pond water samples, affording recoveries of 86–118% with relative standard deviations below 5%. In addition, the method showed acceptable applicability for CIP determination in different pharmaceutical formulations. These results indicate that the Mn-CD-based fluorescent probe provides a convenient, sensitive and promising platform for CIP determination in environmental and pharmaceutical samples.

## 1. Introduction

Ciprofloxacin (CIP) is a widely used second-generation fluoroquinolone antibiotic with broad-spectrum activity and favorable pharmacokinetic properties. It is effective against many Gram-negative bacteria, such as *Escherichia coli*, *Salmonella Typhi* and *Klebsiella pneumoniae*, and also shows activity against some Gram-positive pathogens, including methicillin-susceptible *Staphylococcus aureus* [1]. Since its introduction in the 1980s, CIP has been frequently used in clinical treatment, veterinary medicine and aquaculture to control bacterial infections [2,3]. In clinical settings, it remains an important option for urinary tract, respiratory tract, gastrointestinal, and skin and soft tissue infections [1]. However, CIP is only partially metabolized after administration, and part of the parent compound and its active metabolites can be excreted through urine and feces into municipal wastewater systems, hospital effluents and natural aquatic environments, while pharmaceutical wastewater, agricultural runoff, livestock manure application and aquaculture discharge may further increase its environmental loading and persistence [4,5,6].

Once CIP residues enter aquatic environments or food-related matrices, they may remain active at low concentrations and come into contact with microbial communities. This continuous exposure can alter gut microbial balance, increase the risk of hypersensitivity or other adverse responses and maintain selective pressure on environmental microorganisms [7,8,9]. Such persistent exposure may facilitate the maintenance and dissemination of antibiotic resistance genes, including through horizontal gene transfer [10]. CIP residues can also affect non-target organisms, such as aquatic plants, algae and microorganisms, thereby posing potential risks to ecosystem stability and function [2,11]. These concerns highlight the need for rapid, sensitive, cost-effective and easy-to-operate methods for CIP monitoring in complex environmental and pharmaceutical samples.

Although HPLC and HPLC-MS/MS are widely used for CIP determination and provide reliable quantitative results, their dependence on sample pretreatment, laboratory instruments, skilled operators and data-processing procedures limits their use in rapid field screening and routine monitoring [12,13,14]. These limitations have promoted the development of portable detection strategies for quinolones and CIP, including electrochemical sensors, screen-printed electrodes, smartphone-assisted readout systems, immunoassay-based methods and SERS/MIP-based sensors [15,16,17,18,19]. Among them, ELISA and lateral-flow immunoassays are attractive for screening applications because of their simple operation and relatively high sensitivity. Recent immunoassay studies for fluoroquinolone residues have further improved analytical performance by introducing fluorescence/colorimetric dual-mode readout or colorimetric/SERS bimodal lateral-flow detection combined with machine-learning-assisted data analysis [20,21]. However, immunoassays generally require antibodies or other immunoreagents and their analytical performance may be influenced by reagent stability, cross-reactivity and assay design. In this context, fluorescence sensing based on functional nanomaterials offers an antibody-free alternative, combining rapid optical response, operational simplicity and potential compatibility with visual or portable readout systems [22,23]. Therefore, a low-cost fluorescence method with a low detection limit and reasonable selectivity is still valuable for CIP monitoring in environmental water and pharmaceutical formulations.

Carbon dots (CDs) are ultrasmall fluorescent carbon nanomaterials, generally less than 10 nm in size. They contain sp^2^/sp^3^-hybridized carbon domains and are rich in surface functional groups such as hydroxyl, carboxyl and amino groups, which are closely related to their optical properties and dispersibility [24,25]. Compared with conventional organic fluorophores and semiconductor quantum dots, CDs possess several attractive advantages, including strong and tunable fluorescence emission, high photostability, low photobleaching tendency, good aqueous dispersibility, facile surface functionalization and generally low toxicity with favorable biocompatibility [26,27]. Their optical properties can be effectively regulated by particle size, surface states, precursor composition and heteroatom or metal doping [28,29,30]. In addition, CDs can be readily synthesized from a wide range of inexpensive precursors, including small organic molecules, biomass and other carbon-rich feedstocks, by simple methods such as hydrothermal/solvothermal treatment, microwave-assisted synthesis, and pyrolysis [26,29]. Owing to these merits, CDs have been extensively explored in fluorescence sensing, bioimaging, photocatalysis and drug-delivery-related applications [25,26,27,31].

For antibiotic sensing, CDs can be engineered in different ways to improve fluorescence response, selectivity, and resistance to matrix interference. Heteroatom-doped CDs, such as N- or P-doped CDs, can regulate surface states and electronic structures, thereby improving luminescence behavior and target-induced signal changes. Metal ion-mediated or metal-related CD systems may further introduce coordination-assisted recognition sites for fluoroquinolones. Surface functionalization with ligands, aptamers, or molecularly imprinted polymers can strengthen target selectivity, whereas CD-containing ratiometric systems, including CD/quantum dot, CD/metal nanocluster, and CD/perovskite quantum dot composites, can reduce background fluctuation and enable visual or device-assisted readout. These CD-based design strategies have recently been used for the detection of CIP and other fluoroquinolone antibiotics, supporting the potential of CDs for sensitive, rapid, and practical antibiotic analysis in complex matrices [32,33,34,35].

However, many previously reported CD-based fluorescent platforms for CIP detection mainly rely on noncovalent interactions between CDs and CIP, such as hydrogen bonding, electrostatic interactions and π-π interactions. These interactions may be insufficient to provide strong and specific recognition, thereby limiting the recognition affinity and sensing sensitivity. To address this limitation, a metal-coordination-assisted sensing strategy was introduced by incorporating Mn into CDs. Since CIP contains carbonyl and carboxylate groups in its quinolone structure, these oxygen-containing groups can act as O,O’-bidentate coordination sites for Mn ions. Therefore, Mn incorporation may create coordination-related binding sites that strengthen Mn-CDs-CIP interactions and enhance the fluorescence response. This strategy distinguishes the present Mn-CDs from previously reported CD-based fluorescent probes for CIP detection.

**Scheme 1:**
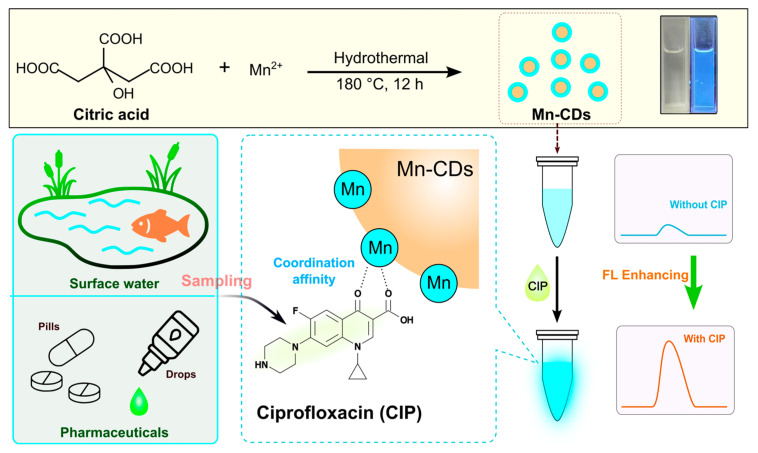
Schematic illustration of the hydrothermal synthesis of Mn-CDs and their coordination-assisted fluorescence sensing mechanism for CIP detection in pond water and pharmaceutical formulations.

In this study, manganese chloride tetrahydrate and anhydrous citric acid were selected as precursors for the hydrothermal synthesis of manganese-doped carbon dots (Mn-CDs). The prepared Mn-CDs were further applied as fluorescent probes for CIP detection in pond water and different pharmaceutical formulations. As illustrated in Scheme 1, Mn incorporation may introduce coordination-related binding sites, allowing CIP to interact with Mn-CDs through its 4-oxo and carboxylate groups. This interaction produced a clear fluorescence enhancement response, and the fluorescence intensity showed a good linear relationship with CIP concentration within the tested range. On this basis, a Mn-CD-based fluorescence method was established for CIP determination in environmental water and pharmaceutical samples.

## 2. Materials and Methods

### 2.1. Reagents and Chemicals

Anhydrous citric acid, manganese chloride tetrahydrate, urea, and CIP were purchased from Greagent (Shanghai, China). Phosphate-buffered saline (PBS), Sodium chloride, potassium chloride, magnesium chloride, calcium chloride, ascorbic acid, glucose, estradiol, uric acid, levofloxacin and norfloxacin were obtained from Adamas (Shanghai, China). Ciprofloxacin hydrochloride tablets (250 mg/tablet) were purchased from Yiling Pharmaceutical (Shijiazhuang, China), ciprofloxacin hydrochloride eye drops (3 mg/mL) from Jucishan Pharmaceutical (Zhengzhou, China) and ciprofloxacin hydrochloride soluble powder (2%) as well as ciprofloxacin lactate injection (50 mg/mL) from Qianfang Animal Health (Hefei, China).

### 2.2. Apparatus and Characterization Instruments

Fluorescence spectra were recorded using an F-4700 fluorescence spectrophotometer (Hitachi, Tokyo, Japan). UV-vis absorption spectra were measured with an N4S UV-visible spectrophotometer (INESA, Shanghai, China). The morphology and microstructure of the samples were characterized using a JEM-F200 field-emission transmission electron microscope (JEOL, Tokyo, Japan). X-ray photoelectron spectroscopy (XPS) analysis was performed using a K-Alpha spectrometer (Thermo Scientific, Waltham, MA, USA). Fourier transform infrared (FT-IR) spectra were recorded on a Nicolet iS20 FT-IR spectrometer (Thermo Fisher Scientific, MA, USA). X-ray diffraction (XRD) patterns were obtained using an Ultima IV X-ray diffractometer (Rigaku, Tokyo, Japan). The pH values were measured with a Five Easy pH meter (Mettler Toledo, Greifensee, Switzerland).

### 2.3. Preparation of Mn-CDs

A total of 2.0 g citric acid and 0.15 g manganese chloride tetrahydrate were added to a beaker containing 30 mL of ultrapure water. The mixture was sonicated for 5 min until completely dissolved, then transferred into a reaction vessel and heated in a drying oven at 180 °C for 10 h. After the reaction vessel was naturally cooled to room temperature, the resulting solution was filtered through a 0.22 μm microporous membrane. After filtration, the solution was placed in a dialysis bag with a molecular weight cut-off of 1000 Da and dialyzed in dilute sulfuric acid solution (pH 2.0) for 12 h. The purified Mn-CDs solution was subsequently collected and stored at 4 °C for later experiments.

### 2.4. Fluorescence Quantum Yield Measurement of Mn-CDs

The fluorescence quantum yield of Mn-CDs was measured by a relative method, with quinine sulfate used as the reference. A quinine sulfate standard solution was prepared in 0.1 M H_2_SO_4_ and all fluorescence measurements were performed at an excitation wavelength of 330 nm. Under the same conditions, quinine sulfate was taken to have a fluorescence quantum yield of 55%.

$$\Phi_{x}=\frac{A_{S}}{A_{x}}\cdot \frac{I_{x}}{I_{s}}\cdot {\left(\frac{n_{x}}{n_{s}}\right)}^{2}\cdot \Phi_{s}$$

The fluorescence quantum yield of Mn-CDs was calculated using the relative method according to the above equation [36]. In this equation, Φ denotes the fluorescence quantum yield, *A* is the absorbance at the excitation wavelength, *I* is the integrated fluorescence intensity collected from 350 to 600 nm under 330 nm excitation, and n is the refractive index of the solvent. The subscripts *x* and *s* refer to the Mn-CDs solution and quinine sulfate standard solution, respectively. To improve the accuracy of the measurement, a series of Mn-CDs solutions and quinine sulfate reference solutions with different concentrations were prepared, and the absorbance of each solution was controlled within the range of 0–0.2.

### 2.5. Fluorescence Sensing Procedure for CIP

The detection method was optimized using a one-factor-at-a-time approach. During optimization, one parameter, such as incubation time or pH, was varied while the other experimental conditions were kept constant. Under the optimized conditions, the CIP detection procedure was as follows. Briefly, 200 μL of CIP standard solution at different concentrations (0, 100, 200, 400, 1000, 2000, 4000, 8000, and 10,000 nM), 700 μL of PBS buffer solution (pH 7.0), and 100 μL of Mn-CDs solution were sequentially added into 2 mL centrifuge tubes. After thorough mixing and incubation for 3 min, the resulting solution was transferred into a 5 mL quartz cuvette for fluorescence measurement. For the blank control, 900 μL of PBS buffer solution and 100 μL of Mn-CDs solution were mixed under the same conditions. The fluorescence intensity was then recorded using a fluorescence spectrometer at the optimal excitation wavelength of 330 nm. To evaluate the contribution of Mn doping to the fluorescence response toward CIP, undoped CDs were used as a negative control. The fluorescence response of undoped CDs toward CIP was measured under the same assay conditions and compared with that of Mn-CDs.

### 2.6. Real Sample Analysis

#### 2.6.1. Pond Water Samples

For the analysis of pond water, 200 μL of pond water sample, 700 μL of PBS buffer solution and 100 μL of Mn-CDs solution were sequentially added into a 2 mL centrifuge tube. After thorough mixing, the fluorescence intensity of the sample was measured at an excitation wavelength of 330 nm. For the recovery test, 50 μL of the prepared CIP standard solution was added to the pond water sample to obtain final CIP concentrations of 200, 400, 800, 900, 1000, and 2000 nM, respectively, and the spiked recoveries were then determined.

#### 2.6.2. Pharmaceutical Samples

For ciprofloxacin hydrochloride tablets, 10 tablets were ground into fine powder and dissolved in water, followed by the addition of 5 mL of dilute hydrochloric acid (0.1 M) to facilitate dissolution. The solution was then made up to 50 mL with water. Before use, the sample solution was further diluted with PBS buffer and mixed thoroughly.

For ciprofloxacin hydrochloride eye drops, 100 μL of the sample was diluted to 1 mL with PBS buffer and mixed well. Subsequently, 100 μL of the diluted solution was further diluted to 10 mL with PBS buffer and mixed thoroughly for later use.

For ciprofloxacin hydrochloride soluble powder, 10 portions were accurately weighed, with masses of 0.1119, 0.0943, 0.1079, 0.0934, 0.1032, 0.0966, 0.0972, 0.1003, 0.0929, and 0.1042 g, respectively. Each portion was dissolved with the aid of 5 mL of dilute hydrochloric acid, then diluted to 50 mL with water, mixed well, and reserved for subsequent analysis.

For ciprofloxacin lactate injection, 10 vials of ciprofloxacin lactate injection (5 mL/vial) were prepared individually and each sample was diluted to 50 mL with PBS buffer. After thorough mixing, 2 μL of each diluted sample was transferred into a 2 mL centrifuge tube and further diluted to 2 mL with PBS buffer for analysis.

For fluorescence measurement, 200 μL of each prepared sample solution, 700 μL of PBS buffer, and 100 μL of Mn-CDs solution were sequentially added into a centrifuge tube. After mixing well, the mixture was incubated for 3 min and then subjected to fluorescence spectroscopic measurement.

## 3. Results and Discussion

### 3.1. Morphological and Structural Characterization of Mn-CDs

**Figure 1:**
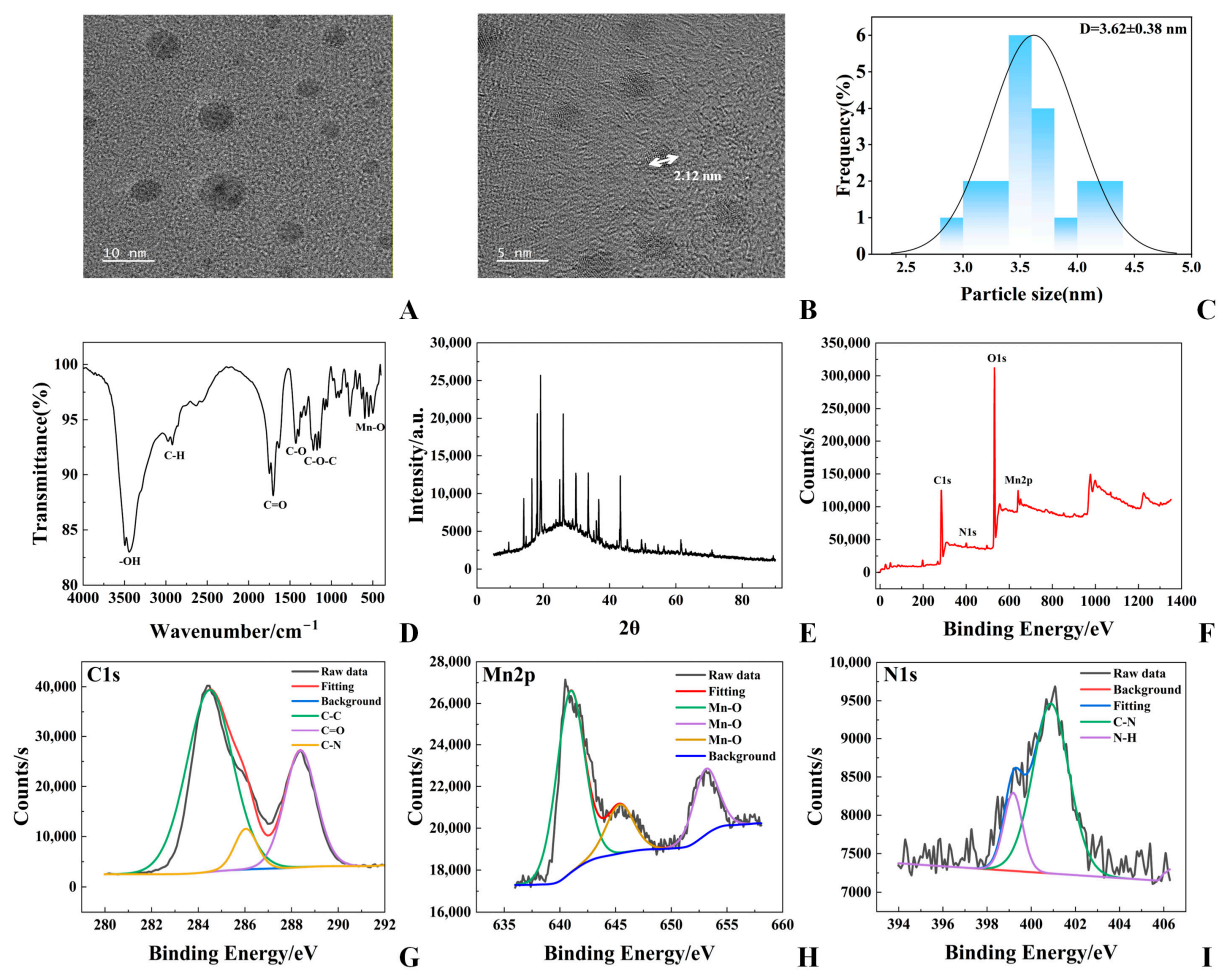
Morphological and structural characterization of Mn-CDs. (**A**) TEM image of Mn-CDs. (**B**) HRTEM image with a lattice fringe spacing of 2.12 nm. (**C**) Particle size distribution of Mn-CDs, with an average diameter of 3.62 ± 0.38 nm. (**D**) FT-IR spectrum of Mn-CDs. (**E**) XRD pattern of Mn-CDs. (**F**) XPS survey spectrum of Mn-CDs. (**G**) High-resolution C 1s spectrum of Mn-CDs. (**H**) High-resolution Mn 2p spectrum of Mn-CDs. (**I**) High-resolution N 1s spectrum of Mn-CDs.

To investigate the morphology and structural characteristics of the Mn-CDs, a series of characterization analyses were performed. The transmission electron microscope (TEM) was used to observe the morphology of the prepared Mn-CDs. The image in Figure 1A shows discrete, nearly spherical nanoparticles with relatively uniform dispersion. According to the particle size histogram in Figure 1C, most Mn-CDs are distributed within 2.50–4.50 nm, giving an average diameter of 3.62 nm.

The fine microstructure of the Mn-CDs was further examined by high-resolution transmission electron microscopy (HRTEM). As shown in Figure 1B, the measured lattice spacing was 2.12 nm, which further confirms the crystalline structure of the Mn-CDs. To further analyze the structural features of the Mn-CDs, XRD analysis was performed, and the result is shown in Figure 1E. The XRD pattern displays a broad diffraction background together with several sharp diffraction peaks, suggesting that the prepared Mn-CDs contain partially crystalline Mn-related species in the carbon-based matrix.

FT-IR spectroscopy was used to further identify the surface functional groups of the Mn-CDs, and the results are presented in Figure 1D. The broad absorption band at 3300–3500 cm^−1^ is attributed to the stretching vibration of O-H. The band in the range of 1500–1680 cm^−1^ corresponds to the stretching vibration of C=O, while the bands observed at 1200–1480 cm^−1^ are assigned to the stretching vibrations of C-N/C-O. In addition, the absorption band around 500 cm^−1^ is attributed to the stretching vibration of Mn-O.

Overall, the above results demonstrate that the prepared Mn-CDs possess a uniform nanoscale morphology and abundant surface functional groups, especially oxygen-containing groups such as hydroxyl, carboxyl and carbonyl groups. These hydrophilic functional groups are beneficial for improving the water solubility and dispersion stability of the Mn-CDs.

To further investigate the elemental composition, chemical states, and surface functional groups of the Mn-CDs, XPS was employed for systematic characterization. As shown in Figure 1F, the Mn-CDs mainly contain four elements, O, C, Mn and N, with atomic percentages of 54.48%, 40.48%, 1.99% and 1.81%, respectively. These results indicate that carbon and oxygen are the major constituent elements, while manganese and nitrogen are present in relatively low amounts as doped elements.

The high-resolution O 1s spectrum in Figure S1 shows a main signal at 531.31 eV, which is related to surface oxygen species on Mn-CDs, including -OH and -COOH groups, together with possible contributions from adsorbed water or lattice oxygen. The presence of these oxygen-containing groups suggests a hydrophilic surface, which is beneficial for the dispersion of Mn-CDs in aqueous solution. In the C 1s spectrum (Figure 1G), the strongest peak appears at 284.80 eV and corresponds mainly to C-C/C=C bonds. Several weaker components at higher binding energies can be assigned to C-O, C=O, and O-C=O bonds. These XPS results are consistent with the FT-IR analysis and support the existence of abundant oxygen-containing functional groups on the Mn-CDs surface.

The Mn 2p spectrum in Figure 1H displays a signal at 640.98 eV, corresponding to the Mn 2p_3_/_2_ level. The binding energy and peak profile suggest that Mn mainly exists in a divalent state and is involved in Mn-O coordination within the Mn-CDs. This assignment agrees with the manganese oxide phase observed in the XRD pattern and the Mn-O vibration detected by FT-IR. As shown in Figure 1I, the N 1s peak at 400.09 eV indicates that nitrogen has been successfully doped into the carbon framework. These N-related sites, possibly associated with pyrrolic N, amino groups or other surface nitrogen species, may influence the electronic structure and provide additional active sites on the Mn-CDs surface.

Overall, the XPS analysis confirms the presence of oxygen-, nitrogen- and manganese-related surface species on the Mn-CDs. These functional components contribute to the aqueous dispersibility of Mn-CDs and may also provide interaction sites for CIP. The rich surface chemistry and Mn-related coordination sites are therefore beneficial for the subsequent fluorescence sensing process.

### 3.2. Optical Properties and Fluorescence Behavior of Mn-CDs

**Figure 2:**
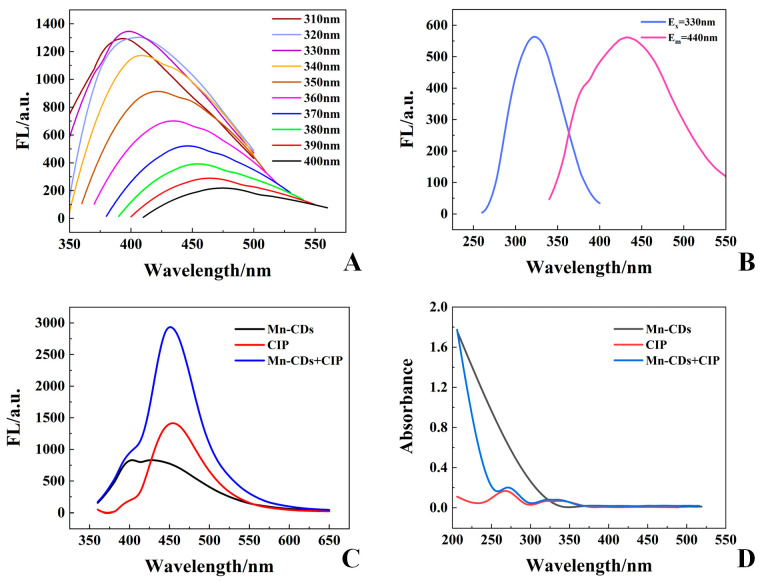
Optical properties of Mn-CDs and their fluorescence response to CIP. (**A**) Excitation-dependent emission spectra of Mn-CDs. (**B**) Fluorescence excitation and emission spectra of Mn-CDs. (**C**) Fluorescence emission spectra of Mn-CDs, CIP, and the Mn-CDs-CIP system. (**D**) UV-Vis absorption spectra of Mn-CDs, CIP, and the Mn-CDs-CIP system.

Emission spectra collected under different excitation wavelengths are presented in Figure 2A. When the excitation wavelength was varied from 300 to 400 nm, the emission peak exhibited an obvious red shift, showing the excitation-dependent fluorescence of Mn-CDs. The strongest emission was obtained at an excitation wavelength of 330 nm, whereas further increasing the excitation wavelength led to a decrease in fluorescence intensity. Consistently, Figure 2B shows a maximum excitation peak at 330 nm and an emission maximum at 440 nm. Therefore, 330 nm was selected for subsequent fluorescence assays.

The response of Mn-CDs to CIP was then examined to assess their sensing capability. After adding 1 μM CIP to the Mn-CDs solution, the emission intensity near 450 nm increased noticeably compared with that of Mn-CDs alone (Figure 2C). This fluorescence enhancement suggests that CIP can effectively modulate the emission behavior of Mn-CDs, providing the basis for constructing a Mn-CD-based fluorescence sensing system. To further evaluate the role of Mn doping, undoped CDs were used as a negative control and tested under the same assay conditions. As shown in Figure S2, the CDs-CIP control exhibited a much weaker fluorescence signal than the Mn-CDs-CIP system, indicating that Mn incorporation contributed to the enhanced fluorescence response toward CIP. In addition, the spectra of CDs, CIP and Mn-CDs alone suggest that the enhanced signal of the Mn-CDs-CIP system cannot be simply attributed to the fluorescence of the individual components.

The UV-Vis absorption spectra of Mn-CDs were compared before and after CIP addition to further clarify the sensing process. As shown in Figure 2D, CIP caused little change in the absorption profile of Mn-CDs. The absence of a new absorption band suggests that no strongly absorbing complex was formed, and the fluorescence enhancement was not mainly associated with an obvious ground-state interaction. Combined with the significant fluorescence enhancement observed in the fluorescence spectra, this effect is presumed to be associated with the promoting role of Mn-CDs in the intrinsic fluorescence emission process of CIP, possibly by enhancing its radiative transition efficiency through mechanisms such as energy transfer, surface-state modulation, or microenvironmental polarity regulation. Using quinine sulfate as the reference, the fluorescence quantum yield of Mn-CDs was determined to be 4.44%. The corresponding linear fitting plots and raw absorbance-integrated fluorescence intensity data are provided in Figure S3 and Table S1, respectively. To further clarify the optical characteristics of the proposed Mn-CDs probe, a brief comparison with other representative optical sensing platforms for antibiotic detection, including lanthanide-based MOFs, UCNPs, plasmonic SPR/LSPR systems and quantum-dot-based probes, is provided in Table S2.

### 3.3. Stability and Optimization of the Mn-CD-Based Fluorescence Sensing System

**Figure 3:**
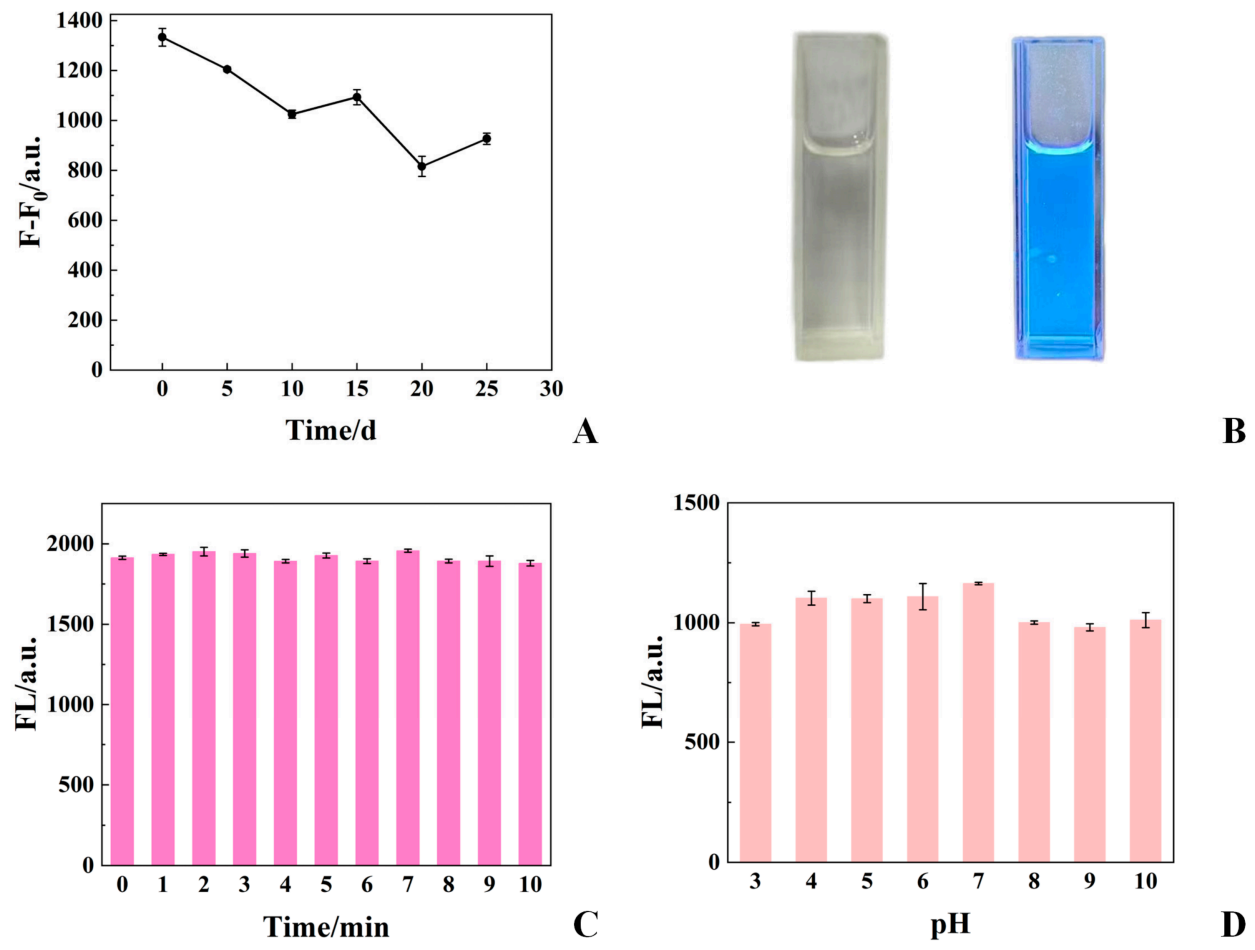
Stability and optimization of the Mn-CD-based fluorescence sensing system for CIP detection. (**A**) Variation in the fluorescence intensity difference ($F-F_{0}$) of the Mn-CDs sensing system during 25 days of storage. (**B**) Digital photographs of the Mn-CDs solution under visible light and UV light, showing bright blue fluorescence under UV irradiation. (**C**) Influence of reaction time on the fluorescence response toward CIP. (**D**) Influence of pH on the fluorescence response toward CIP.

The stability of the Mn-CDs sensing system was followed for 25 days. Mn-CDs dispersed in buffer solution and Mn-CDs mixed with CIP in the same buffer were stored and measured every 5 days, and the difference between their fluorescence intensities was expressed as ($F-F_{0}$). The response decreased gradually during storage, with minor fluctuations between individual time points (Figure 3A). This result suggests that the Mn-CDs retained a measurable fluorescence response to CIP after storage, although the signal weakened over time. The visual fluorescence of Mn-CDs is shown in Figure 3B. The aqueous solution was almost transparent under daylight and displayed bright blue emission when exposed to UV light.

Reaction time and pH were optimized to obtain a stable fluorescence response for CIP detection. Based on preliminary tests, the Mn-CDs volume was fixed at 100 μL, and 700 μL of buffer solution was added to the sensing system. All quantitative data were obtained from three parallel experiments and are presented as mean values to improve the reproducibility and reliability of the results.

The incubation time was first optimized to obtain a rapid and stable fluorescence response. After CIP was introduced into the Mn-CDs solution, the emission intensity changed rapidly and reached a stable level within a short time (Figure 3C). No obvious variation was observed during the subsequent monitoring period, suggesting that the Mn-CDs-CIP system responded quickly under the selected conditions. Based on the rapid signal stabilization and the need for efficient analysis, 3 min was chosen as the incubation time for later experiments.

The fluorescence response of the Mn-CDs-CIP system varied with pH over the range of 5.0–9.0. The signal increased as the solution approached neutral pH and decreased under weakly alkaline conditions, with the strongest response observed at about pH 7.0 (Figure 3D). Based on the above optimization results, the assay was performed using 100 μL of Mn-CDs solution and 700 μL of buffer solution, followed by incubation with CIP for 3 min at approximately pH 7.0.

### 3.4. Selectivity and Analytical Performance for CIP Detection

To systematically evaluate the selectivity of the synthesized Mn-CDs toward CIP and their anti-interference capability in practical analysis, a series of potential interfering substances commonly coexisting with CIP in environmental or biological samples were selected for comparative experiments. These interfering substances included common metabolites (urea, uric acid and glucose), an endogenous hormone (estradiol), an antioxidant (ascorbic acid), common inorganic ions (K^+^, Na^+^, Mg^2^^+^ and Cl^−^) and the structural analogue levofloxacin. In the selectivity experiments, the final concentration of each interfering substance was fixed at 10 μM, whereas the final concentration of CIP and levofloxacin was 1 μM. All fluorescence measurements were carried out under identical experimental conditions to ensure the comparability and reproducibility of the results.

**Figure 4:**
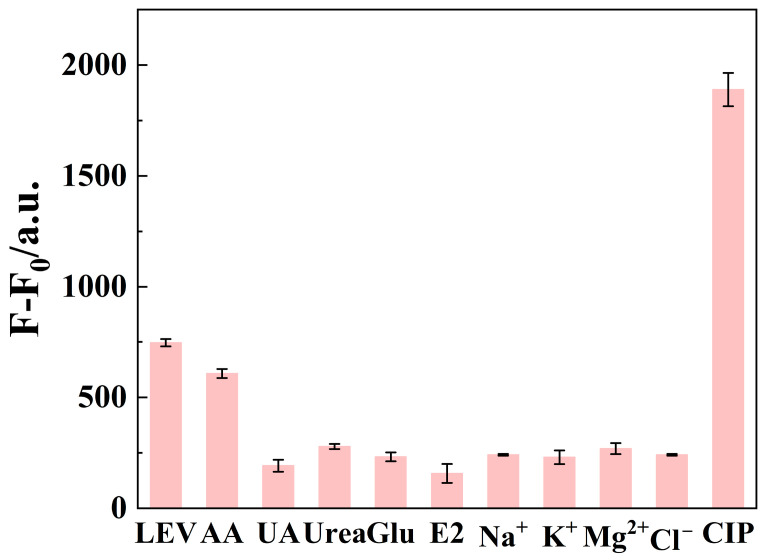
Selectivity of the Mn-CD-based fluorescence probe toward CIP. Fluorescence responses of the Mn-CDs system to CIP and various interfering substances, including levofloxacin (LEV), ascorbic acid (AA), uric acid (UA), urea, glucose (Glu), estradiol (E2), Na^+^, K^+^, Mg^2^^+^ and Cl^−^, expressed as ($F-F_{0}$).

As shown in Figure 4, several interfering substances, particularly ascorbic acid and uric acid, caused a slight enhancement in the background fluorescence of the Mn-CDs system. In addition, hydroxyl- or amino-containing compounds such as glucose and urea also produced limited fluorescence responses. However, the fluorescence enhancement induced by these substances was significantly weaker than that triggered by CIP, indicating that their influence on CIP detection was negligible under the present experimental conditions. Overall, the Mn-CDs fluorescent probe exhibited good selectivity toward CIP and could effectively resist interference from a variety of common coexisting substances, demonstrating its potential applicability in complex sample matrices.

**Figure 5:**
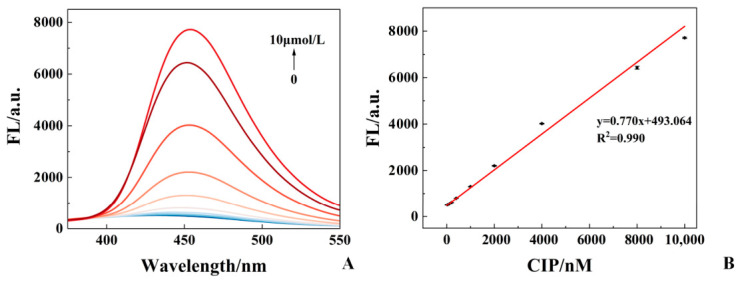
Fluorescence response of the Mn-CD-based sensing system toward CIP and the corresponding calibration curve. (**A**) Fluorescence emission spectra of the Mn-CDs system upon the addition of different concentrations of CIP (0 nM–10 μM). (**B**) Calibration curve for CIP detection based on the fluorescence intensity of the Mn-CDs system.

Based on the optimized experimental conditions, 200 μL of CIP standard solutions at different concentrations were added to centrifuge tubes containing 100 μL of Mn-CDs solution and 700 μL of PBS buffer. After thorough mixing and incubation for 3 min, the fluorescence intensity was measured. As shown in Figure 5, the fluorescence intensity increased gradually with increasing CIP concentration, indicating that the Mn-CDs probe exhibited a concentration-dependent fluorescence response toward CIP. A calibration curve describing the relationship between fluorescence intensity and CIP concentration was established. A good linear relationship was obtained over the CIP concentration range of 20 nM–10 μM, with the regression equation y=0.770x+493.064 and a coefficient of determination R^2^ = 0.990. The adjusted R^2^, MSE and RMSE values were 0.989, 171.108 and 13.081, respectively, further supporting the fitting quality of the calibration model. The limit of detection (LOD) was calculated according to the following equation [37]:

$$LOD=\frac{3\sigma }{K}$$

where σ is the standard deviation of the blank samples and K is the slope of the calibration curve. The blank samples were prepared by mixing Mn-CDs with ultrapure water while keeping all other experimental parameters unchanged, and the standard deviation was obtained from three parallel measurements. The LOD for CIP was calculated to be 1.12 nM, indicating that the Mn-CDs probe can detect CIP at low nanomolar levels in aqueous media. To place this performance in context, the linear range and LOD of the present method were compared with those of recently reported fluorescence-based CIP sensors (Table 1). The Mn-CDs assay covers a linear range of 0.02–10 μM and gives a detection limit comparable to, or lower than, several reported CD-based systems. More importantly, this sensing strategy avoids complex molecular imprinting procedures or dual-emission ratiometric probe construction, while still offering a short response time and reliable sensitivity. Its successful application to pond water and pharmaceutical formulations further supports the practical use of Mn-CDs for CIP analysis.

### 3.5. Application to Pond Water Samples

To further evaluate the practical applicability of the proposed fluorescence sensing method, pond water samples were analyzed through spiked recovery experiments. The results are summarized in Table 2. The recoveries ranged from 86% to 118%, with all relative standard deviations (RSDs) below 5%. These recovery values fall within the commonly used acceptance range of 70–120% for residue-analysis method validation, and the low RSDs indicate good repeatability [42]. These results demonstrate that the developed Mn-CD-based fluorescence sensor is capable of sensitive and quantitative detection of CIP in pond water samples, highlighting its good potential for practical environmental monitoring.

### 3.6. Application to Pharmaceutical Formulations

Different dosage forms of CIP were analyzed, and the measured results were statistically compared with the labeled concentrations indicated on the product packaging. The normality of the data for each dosage form was first assessed. Since the sample size for each group was relatively small (*n* = 10), the Shapiro–Wilk test was employed. The results are presented in Table 3. The data for ciprofloxacin hydrochloride tablets did not follow a normal distribution (*p* < 0.05), whereas the data for the other three dosage forms were normally distributed.

Because the test results for ciprofloxacin hydrochloride tablets did not satisfy the normality distribution, a one-sample Wilcoxon signed-rank test was used to compare the measured values of the 10 samples with the labeled concentration (5 mg/mL). The *p*-value was 0.0507, slightly above the significance threshold of 0.05. Thus, no statistically significant difference was detected between the median measured concentration and the labeled concentration.

For the other three dosage forms, the data followed a normal distribution, and one-sample *t*-tests were used to compare the measured concentrations with the labeled values. The results are listed in Table 4. For ciprofloxacin hydrochloride eye drops labeled as 3 mg/mL, the measured mean differed statistically from the labeled concentration (t(9) = 4.403, *p* = 0.002). The mean difference was 0.245 mg/mL, with a 95% CI of 0.119 to 0.371 mg/mL. The corresponding Cohen’s d value was 1.39, suggesting a large deviation at the statistical level. Nevertheless, the relative deviation of the measured mean was 8.2%, which remained within the ±10% acceptance limit specified in the Chinese Pharmacopoeia. This result suggests that the measured content of the eye drops was still acceptable from a practical quality-control perspective.

For ciprofloxacin hydrochloride soluble powder labeled as 2%, the measured mean was 0.019, close to the labeled value of 0.020. The mean difference was −0.001, with a *p*-value of 0.267 and a 95% CI of −0.003 to 0.001, indicating no statistically significant difference from the labeled concentration. For the ciprofloxacin lactate injection sample labeled as 50 mg/mL, the measured mean concentration was 48.207 mg/mL, corresponding to a mean difference of −1.793 mg/mL. The one-sample *t*-test also showed no statistically significant difference between the measured mean and the labeled value (*p* = 0.594). Taken together, these results support the applicability of the Mn-CD-based fluorescence method for CIP determination in different pharmaceutical formulations.

Beyond the current validation in environmental water and pharmaceutical formulations, the Mn-CD-based sensing strategy may be further developed toward portable detection formats. Although the present method was evaluated using a conventional fluorescence spectrophotometer, its fluorescence enhancement response could be adapted to handheld fluorescence readers, paper-based devices or smartphone-assisted optical readout systems. In future applications, sustainable sensor data management and machine-learning-assisted signal processing may help improve calibration, matrix-effect correction and result interpretation. Artificial intelligence approaches, such as Bayesian regularized artificial neural networks, may also be explored for optimizing assay conditions and improving prediction reliability. These developments could broaden the practical use of Mn-CD-based fluorescence sensing for antibiotic monitoring in environmental and pharmaceutical samples.

## 4. Conclusions

In this study, Mn-CDs were successfully synthesized via a simple one-step hydrothermal method and applied as fluorescent probes for CIP detection. The prepared Mn-CDs exhibited good dispersibility, abundant surface functional groups, uniform nanoscale morphology and favorable fluorescence properties. The incorporation of Mn may provide coordination-related binding sites for CIP, thereby strengthening Mn-CDs-CIP interactions and enhancing the fluorescence response. Under the optimized conditions, the Mn-CD-based sensing system showed a good linear response toward CIP over the concentration range of 20 nM–10 μM, with a detection limit of 1.12 nM. In addition, the proposed method exhibited satisfactory selectivity, good storage stability and acceptable performance in real sample analysis. The method was successfully applied to the determination of CIP in pond water samples and different pharmaceutical formulations. These results indicate that the Mn-CD-based fluorescent probe provides a convenient, sensitive and promising platform for CIP determination in environmental and pharmaceutical samples.

## Figures and Tables

**Table 1:** Comparison of the analytical performance of representative fluorescence-based sensing methods for CIP detection.

Methods	Detection Mode	Linear Range	LOD	Sample Matrix	Ref.
P-CQDs	Fluorescent probe	0.01–0.1 μM	2.06 nM	Milk	[33]
PQDs/CDs@MIP	Ratiometric fluorescence	0.01–30 μM	5.00 nM	Prawn and crucian carp samples	[38]
Al^3^^+^/BSA-CuNCs	Ratiometric fluorescence	0.08–151 μM	30.20 nM	Aquatic foods	[39]
MIPs@O-CDs	IFE-based ratiometric fluorescence	0.08–18 μM	20.70 nM	Milk	[40]
CQDs	Fluorescence quenching	0–40 μM	14.50 nM	Blood serum and artificial urine	[41]
Mn-CDs	Fluorescent probe	0.02–10 μM	1.12 nM	Pond water and pharmaceutical formulations	This study

**Table 2:** Spiked recoveries and RSDs for CIP detection in pond water samples using the Mn-CD-based fluorescence sensor.

Sample	CIP Added (nM)	CIP Found (nM)	Recovery (%)	RSDs (%), *n* = 3
1	200	227.66	113.83	1.53
2	400	423.60	105.90	1.95
3	800	711.28	88.91	0.55
4	900	959.40	106.60	1.29
5	1000	1188.10	118.81	2.46
6	2000	1737.40	86.87	3.37

**Table 3:** Results of the Shapiro–Wilk normality test for different CIP formulations.

Sample	Statistic	df	*p*-Value
Ciprofloxacin hydrochloride tablets	0.676	10	0.000
Ciprofloxacin hydrochloride eye drops	0.905	10	0.246
Ciprofloxacin hydrochloride soluble powder	0.917	10	0.335
Ciprofloxacin lactate injection	0.901	10	0.226

**Table 4:** One-sample *t*-test results for different CIP formulations.

Sample	t	df	*p*-Value	Mean Difference	95% CI of the Difference	
					Upper	Lower
Ciprofloxacin hydrochloride eye drops	4.403	9	0.002	0.245	0.119	0.371
Ciprofloxacin hydrochloride soluble powder	−1.183	9	0.267	−0.001	−0.003	0.001
Ciprofloxacin lactate injection	−0.553	9	0.594	−1.793	−9.132	5.546

## Data Availability

The original contributions presented in this study are included in the article/Supplementary Material. Further inquiries can be directed to the corresponding authors.

## Supplementary Materials

The following supporting information can be downloaded at: https://www.mdpi.com/article/10.3390/bios16070357/s1, Figure S1: High-resolution O 1s spectrum of Mn-CDs; Figure S2: Negative control experiments for evaluating the role of Mn doping in CIP sensing. (A) Fluorescence emission spectra of CDs, CIP and Mn-CDs. (B) Fluorescence emission spectra of CDs-CIP and Mn-CDs-CIP; Figure S3: Linear fitting plots used for fluorescence quantum yield calculation of Mn-CDs. (A) Relationship between absorbance and integrated fluorescence intensity of Mn-CDs. (B) Relationship between absorbance and integrated fluorescence intensity of quinine sulfate used as the reference standard. Table S1: Absorbance and integrated fluorescence intensity data for quantum yield determination of Mn-CDs and quinine sulfate; Table S2: Comparison of representative optical sensing platforms for antibiotic detection. References [38,43,44,45] are cited in the Supplementary Materials.

## Abbreviations

The following abbreviations are used in this manuscript:

CIP	Ciprofloxacin
CDs	Carbon dots
Mn-CDs	Manganese-doped carbon dots
MIP	Molecularly imprinted polymer
