# Supplementary material for: Manganese-Doped Carbon Dots for Sensitive Fluorescence Detection of Ciprofloxacin in Environmental and Pharmaceutical Samples
Source: Biosensors (Basel). 2026 Jun 26;16(7):357. doi: 10.3390/bios16070357 (PMC13406870; doi:10.3390/bios16070357)
Supplement: Supplementary file 1 [file biosensors-16-00357-s001.zip › biosensors-4377073-supplementary.pdf]

biosensors

MDPI

Supplementary Information

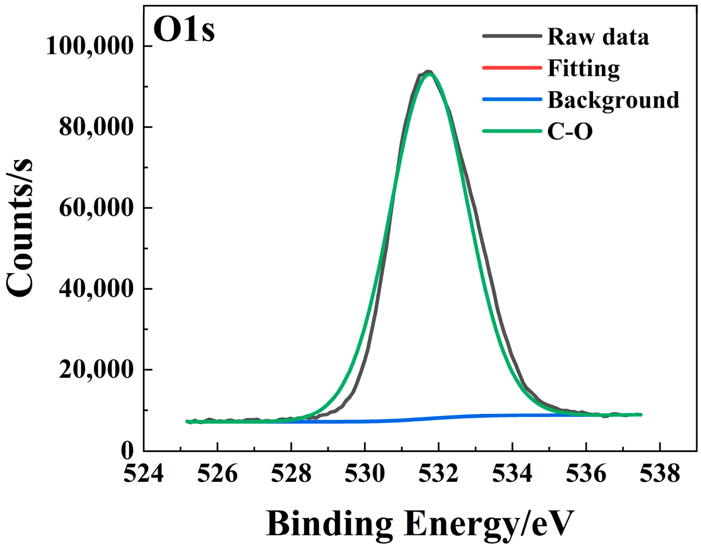

Figure S1. High-resolution O 1s spectrum of Mn-CDs.

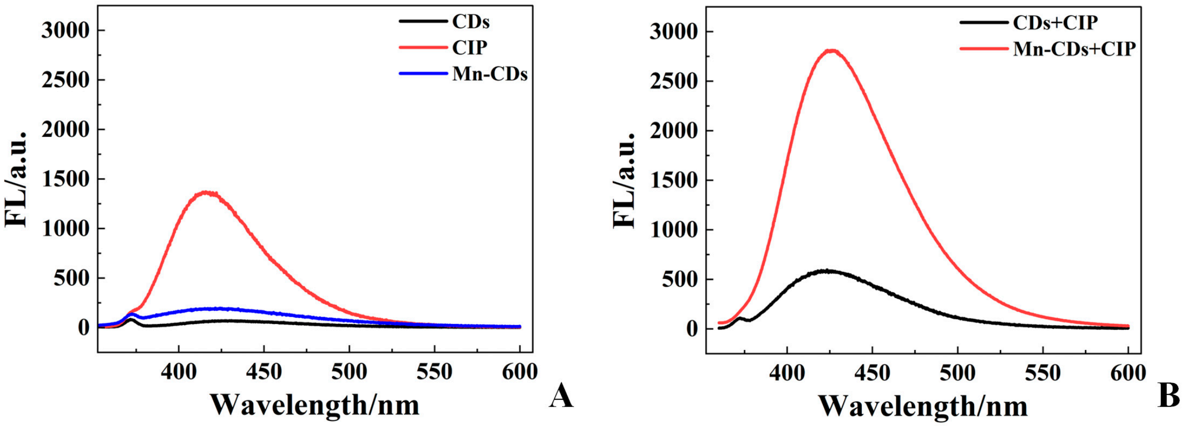

Figure S2. Negative control experiments for evaluating the role of Mn doping in CIP sensing. (**A**) Fluorescence emission spectra of CDs, CIP and Mn-CDs. (**B**) Fluorescence emission spectra of CDs-CIP and Mn-CDs-CIP.

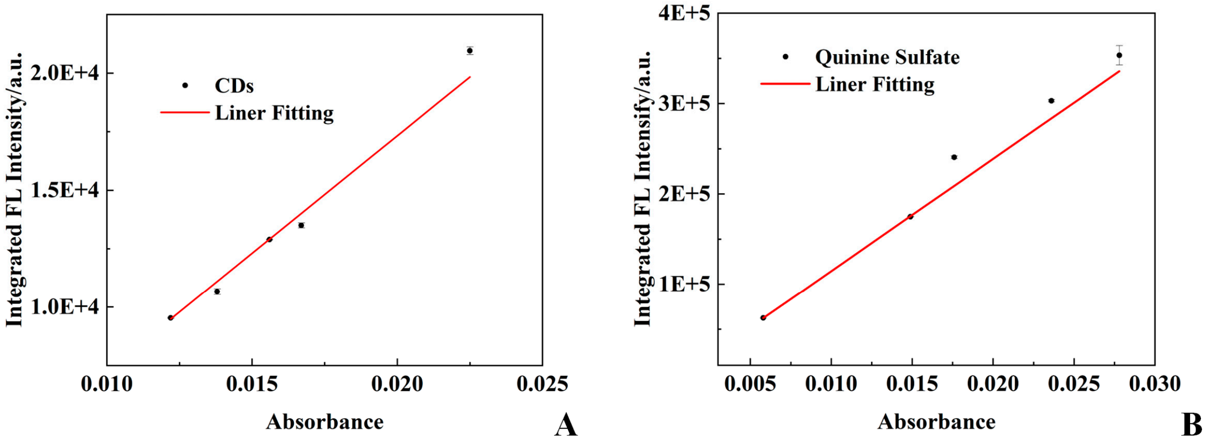

Figure S3. Linear fitting plots used for fluorescence quantum yield calculation of Mn-CDs. (**A**) Relationship between absorbance and integrated fluorescence intensity of Mn-CDs. (**B**) Relationship between absorbance and integrated fluorescence intensity of quinine sulfate used as the reference standard.

Table S1. Absorbance and integrated fluorescence intensity data for quantum yield determination of Mn-CDs and quinine sulfate.

Sample	Absorbance	Integrated fluorescence intensity	Slope	Fluorescence quantum yield
Mn-CDs	0.0122	9.53×10^3	1.00×10^6	4.44%
	0.0138	1.07×10^4		
	0.0156	1.29×10^4		
	0.0167	1.35×10^4		
	0.0255	2.10×10^4		
quinine sulfate	0.0058	6.23×10^4	1.24×10^7	55%
	0.0149	1.75×10^5		
	0.0176	2.40×10^5		
	0.0236	3.03×10^5		
	0.0278	3.53×10^5		

Table S2. Comparison of representative optical sensing platforms for antibiotic detection.

Platform	Optical feature	Advantages	Limitations	Relevance to antibiotic sensing	Ref.
Ln-MOFs	Lanthanide luminescence, sharp emission bands	Long lifetime, large Stokes shift, ratiometric sensing potential	More complex synthesis; stability may depend on solvent and pH	Used as luminescent probes for antibiotic detection with sharp emission and ratiometric response	[1]
UCNPs	Anti-Stokes upconversion emission	Low autofluorescence background, NIR excitation	Relatively low emission efficiency; requires NIR excitation source	Used for low-background antibiotic sensing based on NIR-excited upconversion emission	[2]
Plasmonic SPR/LSPR systems	Refractive-index or plasmonic signal changes	Label-free, real-time detection, signal amplification	Requires controlled nanostructures and optical devices	Used for label-free detection of fluoroquinolone residues, including CIP and ENR.	[3]
PQDs/CDs@MIP	Size-tunable and strong emission	High brightness, multicolor/ratiometric sensing	Potential toxicity or stability concerns for some QDs	Used in fluorescence/ratiometric antibiotic sensing, including PQDs/CDs-based CIP detection.	[4]
CDs/Mn-CDs	Fluorescence emission	Simple synthesis, good water dispersibility, tunable fluorescence	Matrix effects may occur; quantum yield varies with synthesis conditions	Used for rapid fluorescence detection of antibiotics; this work applies Mn-CDs to CIP detection	This study

Reference

1. Chen, S.-Y.; Ye, J.-T.; Wang, L.-Y.; Lin, Y.; Gong, Y.-R.; Xiao, M.-D.; Liu, Y.-F.; Su, Z.-M.; Yang, W.-T. Lanthanide metal–organic Framework Fluorescent Sensor for the Detection of Furazolidone in Tap Water and Animal Feed. *Inorg. Chem.* **2026**, *65*, 5214–5221. https://doi.org/10.1021/acs.inorgchem.6c00129.
2. Su, Q.; Li, J.; Fu, M.; Sun, R.; Chen, J.; Xing, F.; Sun, L. Upconversion/Downshifting Luminescence Ratio Sensor: Single Nanocomposite for Multiple Antibiotic Detection. *Anal. Chem.* **2025**, *97*, 5575–5584. https://doi.org/10.1021/acs.analchem.4c06075.
3. Xu, Q.; Yin, H.; Zhao, Z.; Cui, M.; Huang, R.; Su, R. An Au–Ag@Au fiber surface plasmon resonance sensor for highly sensitive detection of fluoroquinolone residues. *Analyst* **2025**, *150*, 877–886. https://doi.org/10.1039/d4an01162g.
4. Zhang, Q.; Li, X.; Xu, Y.; Xu, Z.; Xu, L. Biomimetic ratiometric fluorescence sensor based on perovskite quantum dots and carbon dots for ultrasensitive ciprofloxacin detection. *J. Hazard. Mater.* **2025**, *490*, 137845. https://doi.org/10.1016/j.jhazmat.2025.137845.

Disclaimer/Publisher's Note: The statements, opinions and data contained in all publications are solely those of the individual author(s) and contributor(s) and not of MDPI and/or the editor(s). MDPI and/or the editor(s) disclaim responsibility for any injury to people or property resulting from any ideas, methods, instructions or products referred to in the content.

https://doi.org/10.3390/bios16070357
